# Population structure and genetic diversity in red clover (*Trifolium pratense* L.) germplasm

**DOI:** 10.1038/s41598-020-64989-z

**Published:** 2020-05-20

**Authors:** Charlotte Jones, Jose De Vega, David Lloyd, Matthew Hegarty, Sarah Ayling, Wayne Powell, Leif Skøt

**Affiliations:** 1 Institute of Biological, Environmental and Rural Sciences, Aberystwyth University, Aberystwyth, Ceredigion United Kingdom; 2 The Earlham Institute, Norwich Research Park, Norwich, Norfolk United Kingdom; 3 Present Address: Sarah Dyer, National Institute of Agricultural Botany, Huntingdon Road, Cambridge, CB3 0LE United Kingdom; 4 Present Address: Scotland Rural College (SRUC), Edinburgh, United Kingdom

**Keywords:** Genetic variation, Plant genetics

## Abstract

Red clover (*Trifolium pratense* L.) is a highly adaptable forage crop for temperate livestock agriculture. Genetic variation can be identified, via molecular techniques, and used to assess diversity among populations that may otherwise be indistinguishable. Here we have used genotyping by sequencing (GBS) to determine the genetic variation and population structure in red clover natural populations from Europe and Asia, and varieties or synthetic populations. Cluster analysis differentiated the collection into four large regional groups: Asia, Iberia, UK, and Central Europe. The five varieties clustered with the geographical area from which they were derived. Two methods (BayeScan and Samβada) were used to search for outlier loci indicating signatures of selection. A total of 60 loci were identified by both methods, but no specific genomic region was highlighted. The rate of decay in linkage disequilibrium was fast, and no significant evidence of any bottlenecks was found. Phenotypic analysis showed that a more prostrate and spreading growth habit was predominantly found among populations from Iberia and the UK. A genome wide association study identified a single nucleotide polymorphism (SNP) located in a homologue of the *VEG2* gene from pea, associated with flowering time. The identification of genetic variation within the natural populations is likely to be useful for enhancing the breeding of red clover in the future.

## Introduction

Red clover is a forage legume, which is used primarily in temperate livestock agriculture. It declined in importance when industrially produced nitrogen fertiliser became available in the early to mid-20th century; but before that, it and other forage legumes were key to maintaining soil fertility, and providing high protein forage for ruminant livestock^1^. However, as we move towards a more sustainable agriculture policy, increased legume use is being encouraged, due to the recognition of their utility^2^. This includes increased production potential, especially in mixtures with grasses; environmentally friendly N input into grassland by virtue of their symbiotic N_2_ fixation; high protein content, and higher voluntary intake and thus better livestock performance^2^.

Red clover is a relatively newly domesticated species, and it was not until around 1000 years ago, that it was first intentionally grown in Southern Spain^1^. Many of the varieties currently on the market in Europe originate from a Mattenklee type of plant, with their growth habit characterised by tall upright stems. However, some natural populations show varied growth habits, characteristically with less dependency on the central crown and a more prostrate nature to stem growth. Under certain temperature and moisture conditions, stems of these prostrate plants are able to produce nodal root growth^3^. The genetics underlying the prostrate growth habit of such red clover ecotypes, as well as the latent disease resistance often associated with natural populations, is of considerable interest for breeding programmes, in terms of furthering the range of climatic and agricultural conditions suited to red clover growth and use^4^. When plants expand into new ecosystems they encounter new and diverse selective pressures^5,6^, which may lead to changes in growth habit, morphology, the partitioning of metabolic resources and disease resistance. This diversity in growth habit has enabled red clover to adapt to varied environmental conditions^7^.

The genetic diversity of natural and cultivated populations of red clover has previously been studied using methods of isoenzymes^8^, AFLP^9,10^ RAPD^11,12^ and SSR markers^13^. All of these studies reported high genetic diversity, and that within population diversity was larger than between populations. It is now possible to obtain large-scale genome wide variation in populations by using next generation sequencing (NGS) technologies. Genotyping by sequencing (GBS)^14^ has become a popular method with which to identify large scale variation in species both with and without a reference genome. The use of GBS has allowed researchers to identify thousands to millions of single nucleotide polymorphism (SNP) markers, which can be used to analyse genetic variation within and between populations, and facilitate the analysis and dissection of complex traits, especially those involved in adaptive selection. While the bioinformatics analysis is more challenging compared to arrays, the cost per SNP is much lower, at least in less intensely studied crop species such as red clover.

The aim of the present work was to assess and analyse the genetic variation present in the accessions of the Genetic Resources Unit at the Institute of Biological, Environmental and Rural Sciences (IBERS). The relatively recent history of red clover cultivation and breeding, would lead to the expectation that differentiation between natural populations and varieties is likely to be small. We report here on the genotyping by GBS of 640 plants of red clover representing 70 natural populations from Europe and Asia and 5 modern varieties. These populations may represent a potential source of novel alleles, which could be used in breeding programmes to alter growth morphology and enhance traits such as disease resistance.

## Results

### GBS data and SNP detection

The sequencing produced an average of 1,700,000 reads per sample (range 203,996 to 5,897,687 of good barcoded reads). From these reads a total of 1,804,668 tags across all 640 plant samples were identified. The SNP analysis in Tassel v5.2, using the red clover genome assembly^15^ as a reference, resulted in 264,927 SNPs across all 640 plants. Substantial filtering of the data (see SNP discovery in the Methods section), resulted in a panel of 12,577 robust polymorphic SNPs. In that process 10 samples were lost due to poor sequencing results. Of the 12,577 SNPs, 8,118 were mapped to the seven chromosomes, and the remaining 4,459 were located in unmapped scaffolds of the assembly. The data presented in the rest of this paper refer to the 8,118 SNPs mapped to the seven chromosomes. The SNPs were identified as transition or transversion SNPs (Supplementary Table S1).

### Phylogenetic relationship

The 8,118 SNPs were used to assess the genetic relationship between the accessions according to the unweighted pair-group method of arithmetic mean (UPGMA) using the package Cluster. The change in slope angle (Supplementary Fig. S1a) indicated that the accessions split into four groups, and the hierarchical UPGMA tree confirmed this (Supplementary Fig. S1b). They consisted of accessions from Asia (including Iran), Central Europe, UK, and the Iberian Peninsula, respectively. Three varieties, Milvus, Britta and AberRuby all belonged to group 2 (Central Europe), and Grasslands Broadway and Crossway to group 4 (Iberian Peninsula), which is consistent with their derivation. The clustering of the accessions revealed two anomalies: the Italian populations Aa4445 and Aa4441 were placed in the clusters represented by Asia and UK, respectively, while all the remaining Italian ecotypes belonged in the Central European group (Supplementary Fig S1).

**Figure 1:**
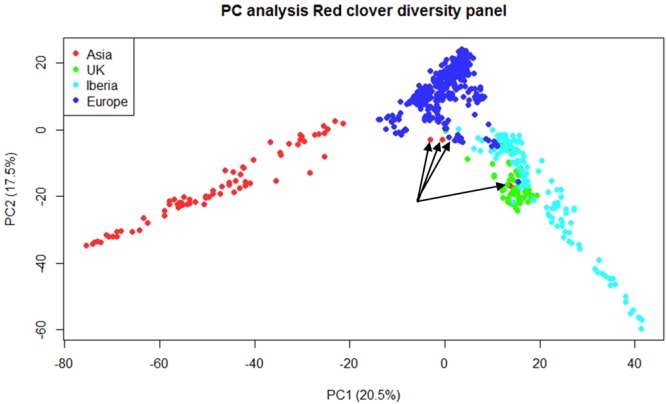
Population structure of the red clover accessions described by Principal Component (PC) analysis. The 4 red dots highlighted by arrows present in the centre of the UK and the Central Europe cluster represent the anomalous Italian accession Aa4445. This accession was described as being part of the Asian group by UPGMA.

**Figure 2:**
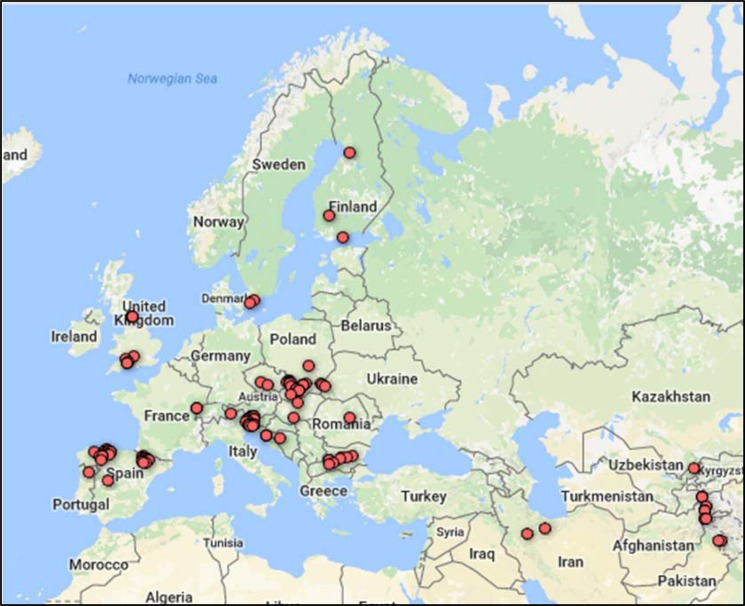
Map of collection sites of the ecotype accessions used for the red clover genetic diversity analysis. The data was accessed through Genesys Global Portal on Plant Genetic Resources, www.genesys-pgr.org, 2018-07-2, filtering for red clover and the holding institute GBR140. The plant genetic resources accession level data was provided by Aberystwyth University Plant Genetic Resources Unit.

A principal component analysis (PCA) of the data was performed. The PCA plot (Fig. 1) illustrates the contribution of the first two principal components to the variation. It demonstrates that the Asian accessions are separate to the European ones, and that the Iberian and UK populations are close to each other, but distinct from those of Central Europe. The PCA indicated that four genotypes of the Italian accession Aa4445 are not part of the Asian cluster as indicated by the UPGMA analysis, but are genetically related to the Central Europe and UK groups (Fig. 1). This is not consistent with the result of the UPGMA analysis (Supplementary Fig. S1). The geographic location of where all the accessions were collected is shown in Fig. 2.

STRUCTURE analysis identified nine ancestral groups in the data as inferred by Δ*K* (Supplementary Fig. S2). STRUCTURE also identified a secondary peak at K = 2, subdividing the population into European and Asian accessions. The nine groups population (Supplementary Fig. S2) could also be classified into four sets, of Asia (group 3), Iberia (group 2 and 8), the UK (group 5), and more loosely Central Europe (group 1, 4, 6, 7, 9). Group 1 consisted mainly of Croatian and Bosnian accessions; group 4 of Italian and Slovakian accessions. Group 7 contained only one accession, that of the Italian Aa4445. Group 9 was a mix of accessions from Hungary, Poland, The Czech Republic and Slovakia. These four sub-groups appeared to be related by geographic distribution. However, group 6, which contained three of the varieties (Milvus, Britta and AberRuby), also consisted of accessions from Northern, Eastern Europe and Italy. There were four oddly placed accessions: Aa4441 from Italy in group 5 (UK), Aa4480 and Aa4487 from Spain and Aa4406 from Pakistan in group 6 (Central Europe). There were four accessions, from Bosnia (Aa4280), Bulgaria (Aa4355, Aa4358) and The Czech Republic (Aa4304), which appeared to consist of mixed populations. Individuals from these accessions belonged to groups 1, 4, 6 and 9 (Supplementary Fig. S2).

### Genetic diversity of the red clover panel

Genetic diversity was evaluated according to the four groups identified by the hierarchical UPGMA analysis. This analysis showed that at the population level, there was more genetic diversity within the accessions than between them, as indicated by the H_S_ and D_ST_ values (Table 1). In the Asian subpopulation the H_O_ was lower than H_E_, while in the three other populations the difference between the two parameters was smaller, or in the case of the UK population, H_O_ was higher than H_E_ (Table 1). This was reflected in the subpopulation inbreeding coefficient, F_IS_, which was only significantly different from zero in the Asian population. The overall F_ST_ value indicated a low to moderate gene differentiation. The F_IT_ value indicated larger deviation from Hardy-Weinberg equilibrium in the total population, than in the individual subpopulations (F_IS_).

**Table 1:** Basic population parameters generated by genetic diversity analysis calculated according to Nei (1977)^61^. The statistical significance of F_IS_ and F_ST_ were derived from χ^2^ tests with one degree of freedom as χ^2^ = NF_IS_^2^, and χ^2^ = 2NF_ST_, respectively; **(*P* < *0.01*); ***(*P* < *0.001*).

	Asia	Central Europe	UK	Iberia	
H_O_	0.208	0.258	0.256	0.251	
H_E_	0.234	0.266	0.246	0.264	
F_IS_	0.104^**^	0.055	−0.010	0.055	
**Overall**					
H_S_	H_T_	D_ST_	F_IS_	F_IT_	F_ST_
0.260	0.275	0.015	0.066	0.136	0.076^***^

Allele frequencies, H_O_ and H_E_ were also calculated for individual accessions. This data revealed no significant differences between the observed and expected levels of heterozygosity, as determined by the Goodness of Fit test (Supplementary Table S2). However, each accession consists of a small number of plants (6-16), so these allele frequencies are not known with high accuracy.

Pairwise F_ST_ values (Table 2) showed that the Asian population was highly differentiated from the UK and Iberian populations, but only moderately differentiated from the Central European population, thus confirming the Asian population as most separated from the three other subpopulations. The UK and Iberian populations were moderately differentiated from each other and to the Central European population. The overall level of gene flow (Nm) was 3.04, and gene flow among accessions within each of the four groups were 1.43 (Asia), 2.13 (UK), 6 (Iberia) and 7.39 (Central Europe).

**Table 2:** Pairwise F_ST_ values calculated in Stampp, between the four groups as defined by UPGMA.

	Asia	Europe	UK	Iberia
Asia	—	—	—	—
Europe	0.111	—	—	—
UK	0.171	0.067	—	—
Iberia	0.166	0.062	0.083	—

Analysis of molecular variance (AMOVA) was performed to assess how much of the total genetic variance could be attributed to different hierarchical levels of division of the total population. The results are summarised in Supplementary Table S3. The first level was among the four groups, Asia, UK, Iberia and Europe. The second level was among accessions within groups, and the third level was individuals within populations. It demonstrates that among group variation accounted for approximately 20% of the total variance, among accessions within groups accounted for 22.9%, and the largest contribution to the variance was within accessions (56.3%).

**Figure 3:**
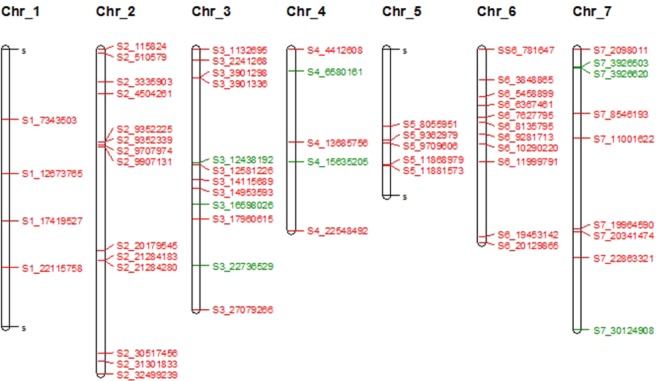
Molecular map showing position of loci potentially under selection as measured by two methods (BayeScan and Samβada). The red SNPs are those that have been identified by both methods. The green SNPs are unique to BayeScan.

Two main methods were used to identify outlier markers in genome scans for selection. BayeScan identified 74 loci and Samβada identified 1,020 loci. They had 60 loci in common spread across the seven chromosomes (Fig. 3). Those loci were found in gene models that have been identified as, amongst others, transcription factors, which are key regulators of gene expression and may have a direct effect on the plants ability to respond to its environment. Others included “household” genes, and some involved in growth responses, flowering time and disease resistance (Supplementary Table S4). In Samβada the 1,020 SNP loci significantly correlated to the geography of the collection sites, after Bonferroni correction for both the Wald and G Score. The majority of the SNPs (85.6% of the 1,020 outliers) were found to be correlated to longitude to some extent (Supplementary Table S5). Regression analysis was performed using the principal components against the geographic co-ordinates. This analysis used all of the SNP data not just the outliers, and a highly significant correlation (r = 0.93) between PC1 and longitude of the collection sites was found (Supplementary Fig. S3). The other two components of latitude and altitude were not significantly correlated.

**Figure 4:**
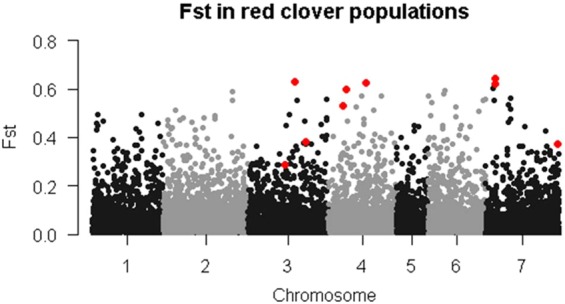
Manhattan plot of average F_ST_ values over the four subpopulations identified in the UPGMA analysis. The markers highlighted in green represent markers with high F_ST_ values and/or of interest because of their putative function. These markers are also highlighted in Supplementary Table S4.

The 60 common loci represent five from intergenic regions, and 55 genic SNPs, of which 51 were assigned gene ontology (GO) terms. GO terms were collected from the genome browser (https://legumeinfo.org) and GO terms analysed in Revigo^16^ to assign biological process, cellular component and molecular function. Over half of these GO terms were assigned to a molecular function (Supplementary Fig. S4). Supplementary Table S4 also highlights 8 markers from 7 genes, five of which have the highest F_ST_ values (Fig. 4). Two of those markers are located on Chromosome 7 in a gene model for a RING/FYVE/PHD type Zinc finger family protein, involved in plant development. The three others are models for a putative methionyl tRNA synthetase on Chromosome 4, an S-adenosyl methionine dependent methyltransferase and a phosphoglycerate kinase protein on Chromosome 3. Three other markers are highlighted because of their potentially interesting function with respect to plant breeding. On Chromosome 3 at position 12,438,192 there is a TIR-NBS-LRR type disease resistance gene, and at position 22,736,529 there is a basic leucine zipper transcription factor, which is a homologue of the flowering time gene *VEG2* in pea^17^. On Chromosome 7 at position 30,124,908 there is a β-D-glucan synthase like gene. Glycans are known to be involved in plant reproduction, especially male gametophyte development^18^.

### Linkage disequilibrium

The average r^2^ for each chromosome was calculated. For the five varieties this value ranged from 0.026 – 0.032 per chromosome, however in the ecotypes this value was lower at 0.007–0.008 (Table 3).

**Table 3:** Summary of LD decay in the varieties and ecotypes. Data collected from LDMap data and Synbreed was used to obtain the average r^2^.

Chromosome	No of markers	Average r^2^	No of markers	Average r^2^
	Varieties		Ecotypes	
1	978	0.026	1197	0.007
2	1212	0.029	1433	0.008
3	1163	0.025	1367	0.007
4	934	0.028	1150	0.008
5	427	0.032	536	0.008
6	820	0.027	963	0.008
7	1004	0.027	1246	0.008

We also compared the decay in LD between the four main groups, and between varieties and ecotypes according to the method of Wang *et al*.^19^, with modifications described^15^. The model excludes values where r^2^ is 0, so the absolute value of the rate of decay is therefore underestimated. In all the comparisons the rate of decay was proportional to population size (Supplementary Fig. S5). Thus, LD in the European population decayed most rapidly, and most slowly in the UK and Asian populations. When we sampled 120 genotypes randomly from the European population of 364, the rate of decay increased to the same level as that of the Asian population, which is of similar size.

### Phenotypic assessment

During the experiment, there was considerable loss of plant material from the first full year to the second year (Supplementary Table S6). This was due to poor winter survival and the inability of many of the ecotypes to survive the cutting regime. The plants were given a lax cut at the end of each year. During the second year, two harvests were collected. The number of plants that survived into year three (2017) was little over 30%, so no further data were collected. This meant that phenotype measurements were collected in one full year only. However, two sets of measurements were collected. From the 514 plants that survived into year two, flowering times were recorded. Of these plants, 415 flowered within the time constraint and a further 99 had flowered by day 100 or later, the day of harvest. There was a large range of variation in flowering across the ecotype panel, with the average being at day 70. Three accessions (Britta, Aa4013 (Denmark) and Aa4038 (Finland)) did not flower before the cut off day. These were all Scandinavian in origin, and Britta was developed as a late flowering variety to maximise vegetative growth^20^. As the data were not normally distributed, it was log_10_ transformed, and subjected to a GWAS analysis using GAPIT (Supplementary Fig. S6). The analysis produced one significant SNP located at Chr3_22736556, which lies within gene 10558. This is a basic leucine zipper transcription factor. A BLAST^21^ search showed it to be homologous to the *VEG2* gene in pea, which is involved in flowering time and inflorescence development^17^. This SNP was also in the list of loci identified as potentially under selection. An investigation into the effects of the alleles at this locus showed that the minor allele reduced the time to flowering by 11 days. The average flowering time for the homozygous major allele was 73.3 days, for heterozygotes it was 66.6 days and for the homozygous minor allele it was 62.4 days. The late flowering accessions Britta, Aa4013 (Denmark) and Aa4038 (Finland) were all homozygous for the major allele for this SNP.

**Figure 5:**
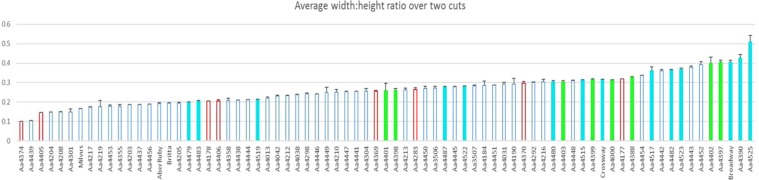
Average plant width to height ratio, with error of the ratio, of phenotypic data collected in year two, and averaged over two cuts. Measurements are in cm, and the graph is coloured in the same way as in the cluster and PC analyses. The varieties are labelled AberRuby, Britta, Grasslands Broadway, Crossway, and Milvus. Average plant heights ranged from 10–153 cm. Average plant widths ranged from 10–44 cm.

In year two of the experiment two sets of measurements were taken for plant height, plant width, and stem number. Over the two collection dates, average plant height per accession ranged between 10–153 cm; the five tallest accessions were in descending order Aa4217 (Slovenia), Aa4444 (Italy), Milvus, Aa4203 (Sweden) and Britta. Average plant width per accession ranged between 10–44 cm; the five widest accessions were in descending order Grasslands Broadway, Aa4443 (Italy), Aa4451 (Italy), Aa4525 (Iberia), and Aa4442 (Italy). To estimate the degree of prostrateness in the population a width:height ratio was calculated. A high ratio described those accessions as being wider than tall, and more prostrate. The ratio ranged between 0.09 – 0.57. The five accessions with the highest ratio were in descending order Aa4525 (Iberia), Aa4402 (UK), Aa4390 (Iberia), Grasslands Broadway, and Aa4523 (Iberia) (Fig. 5, Supplementary Table S7).

The average number of stems per accession was recorded. The values ranged from 1 to 97. The six accessions with the most stems were in descending order Aa4397 (UK), Grasslands Broadway, Aa4399 (UK), Aa4403 (UK), Crossway, and Aa4525 (Iberia) (Supplementary Fig. S7). No significant marker-trait associations were found with the width:height ratio or the stem number.

## Discussion

We present here a study of the genetic diversity and population structure in a collection of *Trifolium pratense* using GBS for the molecular marker data. *Ape*KI was selected as the restriction enzyme, as it is partially methylation sensitive and rarely cuts in retrotransposons. Therefore, *Ape*KI digestion products are fragments preferentially from low-copy genomic regions^14,22^, and are more likely to be genic in origin. This could explain why most of the 60 SNPs potentially under selection were located in transcribed regions (Supplementary Table S4). Red clover is an allogamous plant, and this was reflected in the number of heterozygous biallelic SNPs identified. The 8,118 SNPs were predominantly transitions as opposed to transversions (Supplementary Table S1). This phenomenon has also been reported amongst others in chickpea^23^. The bias towards transitional SNPs is advantageous during natural selection as these SNPs are more likely to conserve protein structure than are transversion SNPs^24^.

The results show a strong relationship between geography and accessions. The germplasm was a collection of material from ecotypes or natural populations from Europe and Asia and five varieties bred from germplasm originating in Europe. There were some anomalies in the genetic structure analysis, and there is some ambiguity as to how many groupings best explain the diversity. According to UPGMA clustering, the change in slope identified most prominently a four group structure, with a minor change in slope angle at two groups. This also was reflected in the PCA (Fig. 1). STRUCTURE identified two groups, but a more likely ancestral origin of nine groups (Supplementary Fig. S2). In all cases, the two group structure separated Asia from Europe; the four group structure differentiated Asia, UK, Iberia and Central Europe, at least partially. The position of the varieties reflected the geographic origin of the germplasm they were bred from. The nine groups identified using STRUCTURE resulted from a subdivision of the Iberian subpopulation into two subgroups, and the Asian subpopulation appeared to be an outgroup from the Central European subpopulation rather than a founder (Supplementary Fig. S2). This subpopulation had the largest heterozygosity deficit of the four, and the highest apparent inbreeding coefficient (Table 1). Given the geographical distribution of the Asian subpopulation in two separate regions, it could be a consequence of the Wahlund effect of “lumping” the two geographically separate groups together. If they do not intercross, it would result in higher H_E_ than H_O_. However, the STRUCTURE and the hierarchical analysis (Supplementary Fig. S2) did not reveal strong evidence of subdivision of the Asian subpopulation, despite the geographically distinct location of the two Iranian and the other Asian populations (Fig. 2).

The samples from the Iberian Peninsula consisted of a moderately unrelated set of genotypes, and the population as a whole was moderately differentiated from the UK and Central Europe (Table 2). At both the local and pan European level, these ecotypes are geographically separated, which may have reduced the potential for gene flow between the populations. However, the Nm value within this group was 6, which suggests some gene flow. The increase in genetic differentiation may be a result of some selection pressure. It is interesting to note that most of the more prostrate or spreading accessions in the panel were from Iberia or the UK (Fig. 5). The potential of prostrate, even stoloniferous red clover for increased grazing tolerance was discussed by Taylor^3^. Likewise, Pecetti^25,26^ reports on the grazing tolerant nature of prostrate alfalfa. Whether grazing pressure has contributed to the prostrate nature of these Iberian accessions is speculative.

The majority of the natural populations, especially from Central Europe and Asia, studied here had an upright nature consistent with the varieties Milvus, Britta and AberRuby (Fig. 5). This growth habit, in natural populations, is likely to occur in habitats with low levels of disturbance^10^. It is also possible that the populations in Central Europe either contain varietal escapes or have undergone considerable gene flow as there was little differentiation between the varieties or the natural populations in either phenotype or genetic diversity (Figs. 1 and 5). This is consistent with the fact that gene flow among the accessions within the European group was highest at 7.39, the largest among the four groups.

The UK population showed a low overall genetic differentiation and a moderate one from Iberia and Central Europe. Island populations have lower genetic diversity when compared to continental populations^27^. This is most likely due to reduced genetic variation in the initial population. The PCA analysis (Fig. 1) and the width to height ratio of plants (Fig. 5) also indicate genetic and phenotypic similarities between the UK and the Iberian subpopulations. To what extent this is a consequence of germplasm exchange between the UK and Iberia, response to similar selection pressures in terms of climatic conditions is difficult to say. The range in geneflow estimates from 1.43 (Asia) to 7.39 (Central Europe) should be enough to prevent genetic drift having an effect^28^. The distribution of allele frequencies among all 75 accessions has a narrow range between 0.6 and 0.78 with a peak around 0.74–0.76. This is consistent with a relatively high Nm value^28^. The only outlier at 0.434 was one accession from the Asian population (Supplementary Table S2), which is also the only population with an inbreeding coefficient (F_IS_) significantly higher than zero (Table 1). The majority of the Asian accessions are from a continental climate with cold winters and dry hot summers (Supplementary Table S8). The climatic and geographic isolation have probably both contributed to the relative genetic differentiation from the other populations (Table 2). The relative importance of climatic conditions and geographical distance in explaining genetic differentiation is difficult to disentangle. The longitudinal correlation is also associated with a climatic gradient ranging from a continental climate in Asia towards a temperate and humid Atlantic climate in Western Europe (Supplementary Table S8).

The overall genetic diversity of this red clover germplasm (H_T_ = 0.275) (Table 1) is comparable to that found in studies using RAPD (H_T_ = 0.29) and isoenzymes (H_T_ = 0.29) in South American, Swiss, and American cultivars respectively^29,30^. Higher values were found with AFLP in Italian Red clover (H_T_ = 0.43)^10^, and allo-enzyme analysis in Caucasian natural populations (H_T_ = 0.35)^8^. Although the data indicated that there was no significant differences between the observed and expected levels of heterozygosity at the individual accession level, they were higher than the subpopulations in this study and H_O_ was higher than H_E_ (Supplementary Table 2). Such excess heterozygosity could be explained by small population size^31^, and is consistent with outbreeding species and indicates a high level of genetic variation within each individual plant and accession, and a negative F_IS_. Red clover has a one locus, gametophytic S-allele system of self-incompatibility, which prevents self-fertilization cross-fertilization by plants that have the same S-allele genotype. It is worth noting that within the Iberian accessions the two varieties Crossway and Broadway had the lowest H_O_ and the 1^st^ and 3^rd^ lowest H_E_. Similarly within the European accessions the three varieties, Britta, Milvus and AberRuby had the 2^nd^, 3^rd^ and 5^th^ lowest H_O_, and the 1^st^, 2^nd^ and 5^th^ lowest H_E_, respectively (Supplementary Table S2). This indicates a slight narrowing of diversity, and selection pressure within the varieties. It should, however be noted that allele frequencies estimated from such small numbers of individuals (6-16) are less accurate.

Red clover is native to Europe, Western Asia and northwest Africa, and is adapted to many edaphic and climatic conditions^7^. The environment, including longitude, latitude and altitude, may result in physiological challenges that in turn may lead to plant morphological and molecular adaptations^32^. Samβada indicated a strong correlation between longitude and adaptive SNPs, and indeed a regression of the first principal coordinate identified the same correlation (Supplementary Fig. S3). These correlations also reflected the population structure as defined by cluster analysis in UPGMA and PCA. The preponderance of prostrate accessions in the Iberian and the UK populations (Fig. 5) meant that there was a weak correlation between longitude and the width:height ratio, but it was not significant (data not shown). Figure 3 shows an outline molecular map of the SNPs potentially under selection, as identified by both BayeScan and Samβada. Although the SNPs cover all seven chromosomes there would appear to be no significant clustering of SNP in any region, especially the 60 under selection in both analyses (red SNPs in Fig. 3). Similar results were reported for winter survivor populations of red clover in Scandinavia^33^.

In addition to BayeScan and Samβada, we also applied a sliding windows Fst scan^34^ to each individual accession. Only one region of interest was identified: In the two Iranian accessions Aa3506 and Aa3507 the same region on Chromosome 7 was identified as potentially under selection (Supplementary Fig. S8). Outlier SNPs of interest in this region included one at position 5,219,303. This SNP is within gene number 38,211, a 7 transmembrane MLO family protein that is involved in mildew resistance^35^. There was another 7 transmembrane MLO family gene very close by. Whether these genes are important for mildew resistance selection remains to be seen.

The AMOVA analysis demonstrated that within population variation (Asia, UK, Iberia, Central Europe grouping) explained most of the variance. This was also evident in the genetic diversity analysis, in which H_S_ was significantly higher than D_ST_. The high heterogeneity and heterozygosity of red clover is expected as already explained by its self incompatibility^36^. Previous studies into the genetic diversity of red clover, using SSR and AFLP markers, have shown that the majority of the diversity is at the within-population level^37–40^. Both the genetic diversity and the AMOVA analyses are thus consistent with previous results.

Domestication and selective breeding would be expected to reduce allelic variation and genetic diversity^41^. Breeding populations are typically small, and by their nature selected for uniformity for traits such as flowering time. It should be borne in mind that red clover breeding is a recent occurrence^7^, and is thus less likely to have had a major effect on allelic variation. LD is strongly dependent on recombination frequency and effective population size^42,43^, so the difference in rate of LD decay seen with the varieties versus the natural populations (Table 3), could be explained by the difference in size of the two populations, rather than any reduction of diversity in the varieties. Supplementary Fig. S6 lends further support to this explanation, as the rate of decay is proportional to the size of the UK, Asian, Iberian and European populations. Obligate outcrossing gives rise to many effective recombination events which causes LD to decay rapidly^44^. The results presented here is consistent with that notion (Supplementary Fig. S5, Table 3). Other outbreeding crop species have also been reported to have low LD, for example in cauliflower^45^ (r^2^ = 0.06) and maize (r^2^ = 0.07)^46^. The results are also in line with what was reported previously for red clover^15^. However, as the extent of LD is very low in the whole panel of accessions used here, interpretation of differences should be treated with caution.

Phenotypic data from all accessions were obtained from the first full season only, due to the high mortality rate in some accessions (Table S6). Mortality could be an interesting phenotype if it was due to lack of adaptation to the climate at IBERS (temperate without dry season, warm summer). However, many confounding factors could have played a role, such as soil and local rhizobia etc. Furthermore, since this work was aimed at identifying promising breeding material, mortality would limit their usefulness in such work. Flowering time is known to be a highly heritable trait, so the single year data are likely to be indicative of the ranking of genotypes. The SNP identified in this analysis was found with high homology to the *VEG2* gene in pea (3^e-61^, 76% identity over 964 bases). The *VEG2* gene is an *FD* homolog that is essential for flowering and compound floral development^17^, and as such is a good candidate for further study and verification in other populations. This gene was also identified as one of the candidates potentially under selection (Supplementary Table S4). The Samβada analysis showed that latitude rather than longitude was the main factor in its variation. This has also been found in other species with genes involved in flowering time responding to environmental cues^47^. It should be noted that although flowering in red clover is promoted by long days, it has no requirement for vernalization^48^.

Vegetative growth analysis were the result of data from two time-points. They showed a variation in growth habit across the panel, especially in terms of width:height ratio (Fig. 5). The Portuguese accession and one of the UK accessions were also among the more prostrate ones (Supplementary Table S7). The prostrate varieties Grasslands Broadway and Crossway are also derived from Iberian populations^49^. They were developed as an alternative to the upright type varieties that dominate the market. Grasslands Broadway was defined as prostrate due to it having the largest width, but in comparison to the two most prostrate accessions included here (Aa4525 and Aa4402) it is twice as tall.

## Conclusions

Based on over 8000 SNP markers, a panel of European and Asian red clover accessions from the IBERS Genetic Resources Unit was divided into four groups according to UPGMA and structure analysis, and the diversity was strongly correlated to longitude. The varieties included in the panel were not distinguishable from the ecotypes in terms of their genetic diversity, and there was no strong evidence for a bottleneck during the breeding process. However, within two Iranian accessions the same region on LG7 was clearly identified as being under selection. Two other methods identified 60 outlier loci indicating signs of selection, but no single chromosomal region was highlighted. The high mortality rate we observed in many of the natural populations is most likely a result of them being unimproved and un-adapted to the growing conditions at IBERS. Nevertheless, this study has shown that some contain novel allelic diversity that could be a source of new variation with potential use in breeding programmes.

## Materials and methods

### Plant material

A total of 75 accessions were used in this work. Of these, 70 were characterised as natural populations or ecotypes, originating from 16 countries in Europe, three from Asia and one from the Middle East (Fig. 2). A further five accessions were commercially available varieties. There were 640 plants in the panel; eight from each ecotype accession and 16 from each of the varieties. The clovers were planted as a spaced plant experiment at IBERS, Gogerddan (52.43° latitude, −4.02° longitude) in 2015 in a randomized block design with 8 replicate plants arranged in 2 blocks with rows of 4 plants per accession in each block.

The countries were allocated to their geographic regions according to The World Factbook and Eurovoc Table (Supplementary Table S8). The climate zones were based on the Köppen-Geiger classification^50^. The five varieties (Supplementary Table S9) were chosen for certain characteristics. AberRuby is an old IBERS variety. It has a lax growth habit, with few stems and its agronomic use has diminished. Crossway and Grasslands Broadway are early generation varieties developed at AgResearch in New Zealand from Portuguese and Spanish ecotype collections. The plants have a creeping growth habit and may under certain damp conditions produce runners that root from the nodes^49^. Milvus and Britta are tall MattenKlee type varieties that were developed in Switzerland and Sweden, respectively. Milvus is an early flowering variety, which has many high yielding stems and was bred for dry hay production. It is also reported to show resistance to *Sclerotinia trifoliorum* (crown rot)^51^. Britta is a late flowering variety^20^ and has a degree of resistance to stem nematode^7^.

### DNA extraction and genotyping by sequencing

DNA was extracted from 100 mg of fresh young leaf tissue using a Qiagen DNeasy extraction kit in a 96 well format. The DNA concentration was measured using a Qubit™2.0, and normalized to 10 ng µl^−1^ with sterile TE buffer. The DNA was prepared for sequencing following the published GBS protocol^14^, with modifications. *Ape*KI was used as the restriction enzyme, and bar coded adapters were annealed to each genotype. 16 of the annealed DNA samples were pooled at a time across the plate, and cleaned with magnetic beads. The concentration of the 16 pooled genotypes (6 per plate) was measured by Qubit™2.0, and 40 ng was used for PCR amplification. The PCR product was cleaned with magnetic beads, and the concentration measured by Qubit™2.0. The PCR amplification was repeated 3 more times. All of the PCR products were then mixed at equal concentration (10 ng µl^−1^) to form the final library, which was analysed in a 96-plex format with 125 bp single end sequencing using an Illumina HiSeq. 2000 NGS platform.

### SNP discovery

TASSEL5v2^52^ and BWA^53^ were used in conjunction with the red clover genome^15^ in a reference based GBS pipeline to identify high quality SNPs within the populations. In the TASSEL5v2 pipeline, the quality of the SNP calling was set with the following parameters: 20 bp minimum and 64 bp maximum length; a Phred quality score of 20 was used ensuring a base call accuracy of 99%, or a 1:100 probability of incorrect base calling. All Phred scores in the sequences were at 30 (99.9%). There was a minimum cut off of 10 reads per SNP site, and the minor allele frequency was set to 0.05 to allow for heterozygote calling. For a SNP to pass into the final count, it had to be present in at least 80% of the genotypes. Finally, missingness was set to 0.1 per genotype. This ensured that genotypes with less than 10% of the SNP were excluded from the dataset. The data were analysed in R studio using a variety of packages^54^. Data re-coding and imputation of missing markers was implemented in the R package Synbreed^55^. The imputation was performed by random sampling from the allele distributions, with the minor allele frequency set to 0.05, and data missingness threshold to 0.2.

### Data analysis

#### Population structure and statistics

Using all of the markers, the population was grouped into respective clusters by using UPGMA in the program Cluster^56^. This package was also used to produce a dendrogram with phylogenetic relationships (termed relationship tree in the text). A principal component analysis (PCA) was performed using R, and coloured according to the derived clusters from the above analysis. To further identify delimitations in the population structure and to infer ancestry, STRUCTURE v2.3^57^ was used with an unbiased Bayesian approach with Markov chain Monte Carlo (MCMC) clustering of samples. The data were assessed for K values ranging from 1 to 15 with burn-in and MCMC iterations set to 20,000 each. For each value of K, three replications were made. STRUCTURE harvester web v0.6.94^58^ was used to find the optimum K value for the population using the Δ*K*. This assigned the accessions to their genetic group. A total of 250 SNP were used per chromosome.

An analysis of molecular variance (AMOVA)^59^ was performed on the data using the R package Pegas^60^. A hierarchical analysis was carried out with three levels: among the four groups identified by the UPGMA analysis, and among accessions within groups, and within accessions.

Once a population structure had been defined according to the UPGMA analysis, the populations were assessed for genetic diversity. The measurements included observed (H_O_) and expected (H_E_) heterozygosity, and the inbreeding coefficient over loci (F_IS_). The genetic differentiation among populations was estimated from the fixation index (F_ST_), the between groups diversity by F_IT_, and the overall genetic diversity H_T_. D_ST_ is the between population genetic diversity, and was obtained by subtracting the average of individual population H_E_ from the total genetic diversity, H_T_. The parameters were obtained according to Nei^61^. The pairwise comparison of F_ST_ was obtained using the R programme StAMPP^62^.

#### Outlier detection

To identify candidate loci potentially influenced by selection, three methods were used: F_ST_ genome scan^34^, BayeScan v2.0^63^ and Samβada v0.5.3^64–66^. To test the effect of environment (*i.e*. geographic origin) on genetic diversity, the geographical coordinates were used for each sampling site. Samβada is a spatial correlation method, which uses non-random associations between cause and effect. It uses Moran’s I correlation^67^ which assesses the overall clustering of the data, and this is related to the first law of geography “everything is related to everything else”^68^. Parameters were set to spatial for the autocorrelation, and nearest neighbours 20 for the weighting. Significant loci were identified after Wald and G tests following Bonferoni correction at a 99% confidence level. Significant outlier SNPs that were common to all three analyses were identified in the genome assembly, and gene models assessed using BLAST2GO^69^ for biological and molecular processes, and cellular component, and to assign gene ontology (GO term).

#### Linkage disequilibrium

LD was calculated as the squared allele frequency (r^2^) between each pair of SNP loci. Alleles were only considered if the minor allele frequency was above 0.05, as r^2^ has large variances if rare alleles are considered^70^. Modelling of LD decay was performed using a custom R script derived from the method described in Wang *et al*.^19^, and modified as described^71^.

### Phenotype measurements

Flowering time was recorded per plant from the initial start point of 1^st^ April until 21^st^ June in 2016 (day 1–81) and a further date recorded as day 100 (10^th^ July 2016) as day of harvest. Measurements for plant height, width, number of stems and two harvest wet weights were also recorded. Any data that were not normally distributed were log transformed to improve homogeneity and analysed in Genome Association and Prediction Integrated Tool (GAPIT) for GWAS. The analysis was performed with the compressed mixed linear model^72^ implemented in the GAPIT R package^73^.

## Supplementary information

Supplementary Information.

Supplementary Information2.

## Data Availability

The genotypic data have been deposited as raw sequence reads in the NCBI database under BioProject PRJEB30826 and phenotypic data are available upon request from the corresponding author.
