# Supplementary material for: Population structure and genetic diversity in red clover (Trifolium pratense L.) germplasm
Source: Sci Rep. 2020 May 20;10:8364. doi: 10.1038/s41598-020-64989-z (PMC7239897; doi:10.1038/s41598-020-64989-z)

Population structure and genetic diversity in red clover (*Trifolium pratense* L.) germplasm

Charlotte Jones[1], Jose De Vega[2], David Lloyd[1], Matthew Hegarty[1], Sarah Ayling[2,3], Wayne Powell[1,4] and Leif Skøt[1*]

[1]Institute of Biological, Environmental and Rural Sciences, Aberystwyth University, Aberystwyth, Ceredigion, United Kingdom

[2]The Earlham Institute, Norwich Research Park, Norwich, Norfolk, United Kingdom

[3]Current contact details: Sarah Dyer, National Institute of Agricultural Botany, Huntingdon Road, Cambridge CB3 0LE, United Kingdom

[4]Current address: Scotland Rural College (SRUC), Edinburgh, United Kingdom

[*]Corresponding author

E-mail: lfs@aber.ac.uk

Supplementary Material

- Tables S1-S9
- Figures S1-S8

Table S1. Number of transversion and transition SNP, identified in the entire population.

SNP type	Total population
Transversion	3119
Transition	4998

Table S2. Allele frequencies, expected and observed heterozygosity for each of the 75 accessions of the red clover panel. χ^2 p-values for Ho vs He Goodness of fit test are given for each of the four groups.

Population	Genotypes	Allele frq	Ho	He
**Asia**	76			χ^2 p-value = 0.30
Aa3506	7	0.704	0.328	0.312
Aa3507	8	0.665	0.365	0.325
Aa4177	7	0.705	0.332	0.331
Aa4178	8	0.434	0.310	0.260
Aa4283	7	0.645	0.444	0.352
Aa4369	7	0.635	0.363	0.322
Aa4370	8	0.713	0.284	0.302
Aa4374	8	0.668	0.174	0.255
Aa4405	8	0.659	0.343	0.303
Aa4406	8	0.721	0.508	0.343
Average		0.655	0.345	0.310
**UK**	64			χ^2 p-value = 0.85
Aa4000	8	0.740	0.324	0.309
Aa4388	8	0.714	0.350	0.319
Aa4397	8	0.697	0.364	0.320
Aa4398	8	0.663	0.394	0.340
Aa4399	8	0.735	0.350	0.315
Aa4401	8	0.721	0.498	0.342

Aa4402	8	0.748	0.327	0.306
Aa4403	8	0.693	0.396	0.334
Average		0.714	0.375	0.323
**Iberia**	126			χ^2 p-value = 0.99
Aa4390	8	0.613	0.384	0.347
Aa4479	8	0.664	0.413	0.349
Aa4480	8	0.741	0.351	0.315
Aa4482	8	0.721	0.360	0.311
Aa4483	8	0.721	0.364	0.325
Aa4487	8	0.748	0.350	0.316
Aa4515	8	0.720	0.489	0.347
Aa4517	8	0.671	0.357	0.313
Aa4519	8	0.718	0.359	0.328
Aa4522	8	0.754	0.344	0.312
Aa4523	8	0.715	0.344	0.319
Aa4525	8	0.602	0.348	0.305
Aa4939	15	0.744	0.333	0.310
Aa4940	15	0.750	0.328	0.299
Average		0.706	0.366	0.321
**Europe**	364			χ^2 p-value = 0.99
Aa4013	8	0.716	0.376	0.324
Aa4031	8	0.775	0.327	0.301
Aa4038	8	0.745	0.350	0.319
Aa4042	8	0.749	0.350	0.313
Aa4184	8	0.705	0.367	0.329
Aa4190	8	0.756	0.345	0.313
Aa4203	8	0.712	0.358	0.314
Aa4204	8	0.740	0.262	0.289
Aa4205	8	0.758	0.346	0.310
Aa4208	8	0.739	0.352	0.316
Aa4210	8	0.743	0.366	0.318
Aa4212	8	0.738	0.347	0.317
Aa4213	8	0.686	0.391	0.324
Aa4216	8	0.761	0.343	0.310
Aa4217	8	0.748	0.350	0.310
Aa4219	8	0.751	0.351	0.307
Aa4292	8	0.757	0.341	0.311
Aa4298	8	0.754	0.332	0.308
Aa4301	8	0.719	0.362	0.316
Aa4304	8	0.758	0.339	0.307
Aa4355	8	0.744	0.313	0.314
Aa4358	8	0.724	0.364	0.327
Aa4379 (Britta)	16	0.778	0.306	0.287

Aa4437	8	0.749	0.343	0.310
Aa4438	8	0.744	0.357	0.318
Aa4439	8	0.748	0.365	0.312
Aa4441	8	0.722	0.511	0.348
Aa4442	8	0.744	0.353	0.318
Aa4443	7	0.717	0.386	0.332
Aa4444	8	0.739	0.362	0.322
Aa4445	8	0.696	0.372	0.328
Aa4446	8	0.718	0.529	0.355
Aa4447	7	0.749	0.361	0.319
Aa4448	8	0.753	0.348	0.312
Aa4449	8	0.739	0.368	0.321
Aa4450	8	0.744	0.355	0.318
Aa4451	6	0.722	0.381	0.332
Aa4452	8	0.742	0.349	0.311
Aa4453	8	0.752	0.337	0.309
Aa4454	8	0.756	0.344	0.313
Aa4456	8	0.764	0.348	0.307
Aa4554	16	0.780	0.307	0.287
S543	16	0.754	0.325	0.301
Average		0.742	0.357	0.315

Table S3. Summary of Analysis of Molecular Variance (AMOVA) assuming a hierarchical division into four groups consisting of Asia, UK, Iberia and Europe as seen in the UPGMA analysis, then between accessions within groups, and between individuals within accessions. The analysis was carried out using the R package pegas. SSD: Sum of Squares Deviation; MSD: Mean Squares Deviation; df: degrees of freedom; Variance: variance components. ***(*P* < 0.001).

	**SSD**	**df**	**MSD**	**Variance**	**% variation**
Among groups	10.0913	3	3.3637	$\sigma_g^2 = 0.024$	20.7***
Among accessions within groups	20.7010	71	0.2916	$\sigma_p^2 = 0.026$	22.9***
Within accessions	36.6742	555	0.0661	$\sigma_a^2 = 0.066$	56.3***
Total	67.1665	629	0.1072	$\sigma^2 = 0.107$	

Table S4. Position, F_{ST}, GO term, putative gene function and characterisation of the 60 loci identified by both BayeScan and Samβada as outliers and thus potentially under selection (MS Excel file).

Table S5. Number of SNP identified to be under the selection due to geography as determined in Samβada.

Geography	Number of SNP	Percentage (%)
Altitude	33	3.2
Latitude	58	5.1
Longitude	420	41.2
Altitude and latitude	57	5.6
Altitude and longitude	77	7.5
Latitude and longitude	39	3.8
All three	337	33

Table S6. Plant mortality during the experiment.

Year	Start of year	Number died	End of year	Reason
1 (2015)	640	10	630	Poor growth
2 (2016)	514	126 + 115	399	Did not survive winter + the harvesting regime
3 (2017)	200	199		Did not survive winter. Experiment finished

Table S7. The five accessions with the highest width to height ratio. Numbers in brackets are the standard errors of the samples.

Country	Accession	Height (cm)	Width (cm)	W:H ratio	Stem count
Spain	Aa4525	65.7 (± 5.4)	33.4 (±3.3)	0.57 (± 0.34)	65 (± 9)
UK	Aa4402	56.7 (± 6.9)	22.7 (± 2.7)	0.46 (± 0.32)	66 (± 20)
Portugal	Aa4390	74.1 (± 6.3)	31.5 (±3.1)	0.45 (± 0.19)	45 (± 11)
Spain	Broadway	108.9 (± 6.4)	44.0 (±3.6)	0.42 (± 0.10)	85 (± 16)
Spain	Aa4523	86.1 (± 5.7)	31.7 (± 3.2)	0.38 (± 0.06)	34 (± 11)

Table S8. The accession numbers and countries of origin of the ecotypes used in the diversity study of red clover. The eight geographic regions according to The World Factbook and Eurovoc. (ME: Middle East, CA: Central Asia, SA: Southern Asia, EE: Eastern Europe, NE: northern Europe, SE: Southern Europe, SCE: South Central Europe, SWE: South Western Europe). ClimateZ are climate zones based on the Köppen-Geiger classification (see Materials and Methods section). Csa: Temperate dry hot summer; Dsa: Cold dry hot summer; Dsc: Cold dry cold summer; Cfa: Temperate without dry season hot summer; Cfb: Temperate without dry season warm summer; Dfb: Cold without dry season warm summer; ET: Tundra-like; Dfc: Cold without dry season cold summer; Csb: Temperate dry warm summer.

Accession	Country	Region	Latitude	Longitude	Altitude	ClimateZ
Aa3506	Iran	ME	35.7571	50.8992	810	Csa
Aa3507	Iran	ME	35.2185	48.7168	1670	Dsa
Aa4177	Uzbekistan	CA	41.7667	70.1333	1110	Dsc
Aa4178	Uzbekistan	CA	41.5200	70.1800	1170	Dsc
Aa4283	Tajikistan	CA	40.5200	70.4600	840	Dsa
Aa4369	Tajikistan	CA	36.7500	71.6000	2800	Dsc
Aa4370	Tajikistan	CA	36.7167	71.6333	2800	Dsc
Aa4374	Tajikistan	CA	38.0000	71.7500	2200	Dsc
Aa4405	Pakistan	SA	34.3886	73.3001	2200	Cfa
Aa4406	Pakistan	SA	34.3897	73.5696	2400	Cfb
Aa4031	Romania	EE	46.8642	23.5371	475	Dfb
Aa4042	Hungary	EE	46.3936	18.6580	90	Cfb
Aa4184	Poland	EE	49.2333	22.3333	675	Dfb
Aa4190	Poland	EE	50.8500	20.6500	300	Cfb
Aa4292	Czech Rep	EE	49.4833	18.1000	460	Dfb
Aa4304	Czech Rep	EE	49.5500	14.4167	650	Cfb
Aa4298	Slovakia	EE	49.1833	18.2167	380	Dfb
Aa4301	Slovakia	EE	48.8667	19.0333	1269	Dfb
Aa4355	Bulgaria	EE	42.5833	23.2500	1690	Cfb
Aa4358	Bulgaria	EE	42.2667	23.6667	960	Dfb
Aa4013	Denmark	NE	55.7831	11.2895	41	Cfb
Aa4038	Finland	NE	60.1730	24.9436	7	Dfb
Aa4203	Sweden	NE	55.6727	13.0819	12	Cfb
Aa4204	Sweden	NE	55.8506	13.4667	68	Cfb
Aa4000	UK	NE	51.3833	-2.9667	30	Cfb
Aa4388	UK	NE	51.6392	-1.8575	80	Cfb
Aa4397	UK	NE	51.0167	-2.8667	30	Cfb
Aa4398	UK	NE	54.6833	-2.2667	440	Cfb
Aa4399	UK	NE	51.0667	-2.8333	100	Cfb
Aa4401	UK	NE	51.2333	-2.6833	240	Cfb
Aa4402	UK	NE	54.6500	-2.1500	280	Cfb
Aa4403	UK	NE	54.6333	-2.0833	300	Cfb
Aa4205	Croatia	SE	45.4700	15.5800	400	Cfb
Aa4208	Croatia	SE	44.7833	15.0667	1090	Cfb

Aa4210	Bosnia	SE	43.6833	17.6833	500	Dfb
Aa4212	Bosnia	SE	44.1000	17.1400	1180	Cfb
Aa4213	Bosnia	SE	44.0500	16.9167	920	Cfb
Aa4216	Bosnia	SE	44.6000	16.8333	510	Cfb
Aa4217	Slovenia	SE	45.8333	14.2000	850	Cfb
Aa4219	Slovenia	SE	46.3500	13.4833	1400	Cfb
Aa4437	Italy	SCE	46.7667	10.5333	1510	ET
Aa4438	Italy	SCE	46.1000	12.9000	100	Cfb
Aa4439	Italy	SCE	46.1500	12.6000	980	Cfb
Aa4441	Italy	SCE	46.3333	12.5833	930	Cfb
Aa4442	Italy	SCE	45.8500	13.5000	100	Cfb
Aa4443	Italy	SCE	45.8500	13.5167	150	Cfb
Aa4444	Italy	SCE	46.0167	12.4833	100	Dfc
Aa4445	Italy	SCE	46.0500	12.8000	150	Cfb
Aa4446	Italy	SCE	45.8333	12.8000	100	Cfa
Aa4447	Italy	SCE	45.7333	13.4333	15	Cfa
Aa4448	Italy	SCE	45.7667	13.0000	50	Cfa
Aa4449	Italy	SCE	46.5000	13.3667	700	Cfb
Aa4450	Italy	SCE	46.5333	13.4500	1200	Dfc
Aa4451	Italy	SCE	46.5000	13.6000	800	Dfc
Aa4452	Italy	SCE	46.5500	13.1667	1550	Dfc
Aa4453	Italy	SCE	46.5667	13.1000	1050	Dfc
Aa4454	Italy	SCE	46.5000	13.1167	700	Cfb
Aa4456	Italy	SCE	45.8500	12.8000	50	Cfa
Aa4390	Portugal	SWE	41.3333	-7.7667	753	Csb
Aa4479	Spain	SWE	42.7211	-0.3015	1092	Cfb
Aa4480	Spain	SWE	42.7709	-0.8759	1031	Cfb
Aa4483	Spain	SWE	42.7281	-0.5959	1370	Cfb
Aa4487	Spain	SWE	43.3277	-4.8786	347	Cfb
Aa4515	Spain	SWE	42.9835	-5.9416	1495	Csb
Aa4517	Spain	SWE	42.9128	-5.9247	1100	Csb
Aa4519	Spain	SWE	43.3454	-7.0126	894	Csb
Aa4522	Spain	SWE	42.5814	-6.2225	1194	Csb
Aa4523	Spain	SWE	42.5814	-6.2225	1194	Csb
Aa4525	Spain	SWE	43.3790	-5.8658	374	Csb
Aa4482	Spain	SWE	42.9297	-0.6515	944	Cfb

Table S9. The five varieties used in the ecotype panel. The table shows the place of varietal development and the European region according to Eurovoc of the plant material (see table 1 for key to regions).

Variety	Accession	Place of development	European region
AberRuby	S543	IBERS, UK	NE
Britta	Aa4379	Uppsala, Sweden	NE
Broadway	Aa4940	Ag-Research New Zealand	SWE
Crossway	Aa4939	Ag-Research New Zealand	SWE
Milvus	Aa4554	Agroscope, Switzerland	SCE

Figure S1. Population structure of the ecotype panel using cluster analysis. **a**. The change in slope of the graph indicated the most probable number of groups in the population *i.e.* 4 groups. There is also a minor change in slope angle at 2 groups. **b**. Relationship tree, showing all p-values below 0.05 cut-off (FWER corrected). The four groups are coloured to indicate which groups they belong to. **c**. The four regions accounted for in the clustering of the data according to UPGMA, and the number of accession per cluster.

a.

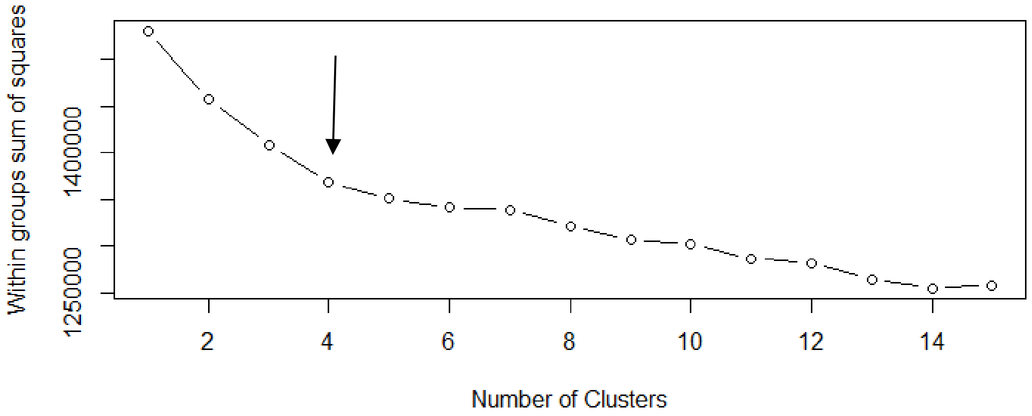

b.

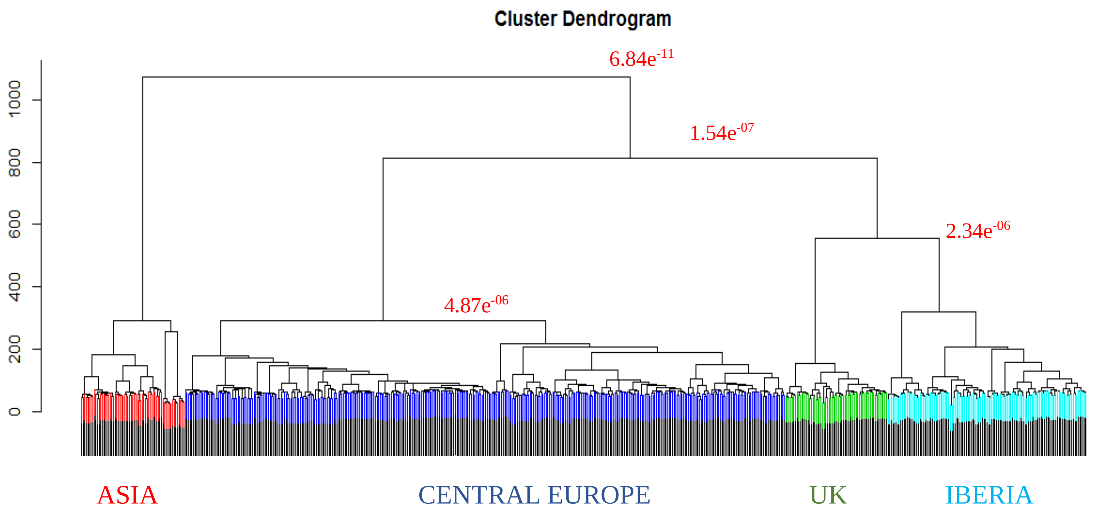

c.

Group	Cluster region	Number of accession
1	Asia + Aa4445	11
2	Central Europe + varieties (Milvus, Britta, AberRuby)	41
3	UK + Aa4441	9
4	Iberian Peninsula + varieties (Grasslands Broadway, Crossway)	14

Figure S2. Population structure of the ecotype panel using STRUCTURE. **a.** STRUCTURE harvester DeltaK plot, indicating major peak at k=9 and a large secondary peak at k=2. **b**. Tree plot of k=9, with the Asian accessions defined as population 3. The European accessions were well defined for The Iberian Peninsula (populations 2 and 8), and the UK (population 5); the remaining accessions however were less defined and grouped into populations 1, 4, 6, 7 and 9. All populations are marked with the same colours as in S2a. **c.** The nine inferred ancestry groups according to STRUCTURE analysis with the accessions and respective countries allocated to each group according to the Q matrix. The table is coloured in accordance with the four group structure as described in 2Sa. The accessions that are thought to have mixed ancestry are coloured in violet, and those that do not cluster with the correct geographical group are coloured in orange. These odd placed accessions are most likely to be introduced or escaped varieties of the Mattenklee type (Milvus, Britta and AberRuby).

a. **b.**

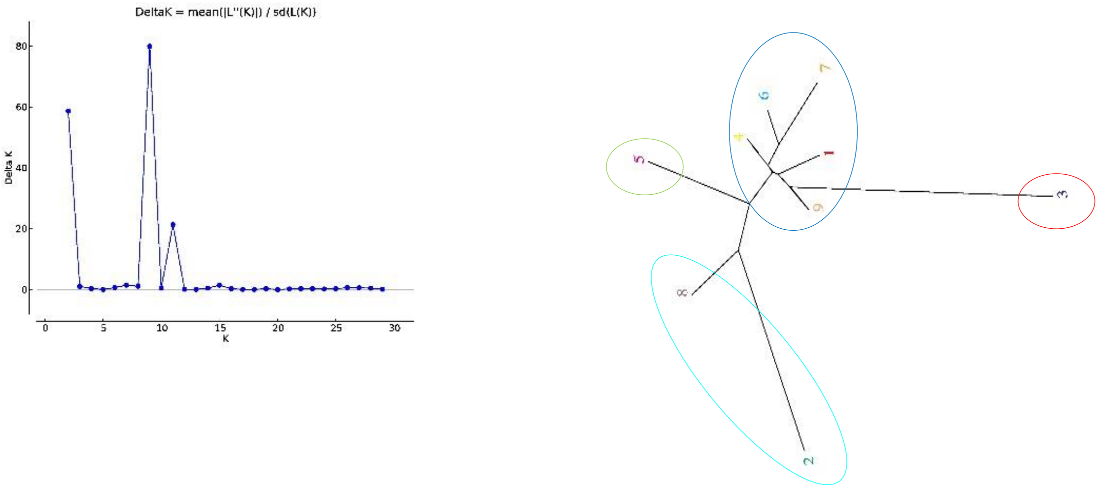

c.

STRUCTURE Group	1		2		3		4		5		6		7		8		9	
Region	Europe 1		Iberia 1		Asia		Europe 2		UK		Europe 3		Europe 4		Iberia 2		Europe 5	
	Country	Accession	Country	Accession	Country	Accession	Country	Accession	Country	Accession	Country	Accession	Country	Accession	Country	Accession	Country	Accession
	Croatia	Aa4208	Portugal	Aa4390	Iran	Aa3506	Slovakia	Aa4219	UK	Aa4000	Varieties	S543	italy	Aa4445	Varieties	Aa4939	Hungary	Aa4042
	Bosnia	Aa4212	Spain	Aa4482		Aa3507	Italy	Aa4439		Aa4388		Aa4379				Aa4940	Poland	Aa4184
		Aa4213		Aa4483	Uzbecistan	Aa4417		Aa4449		Aa4397		Aa4554			Spain	Aa4479	Czech Rep.	Aa4292
	Bosnia	Aa4280		Aa4517		Aa4418		Aa4450		Aa4398	Denmark	Aa4013				Aa4515	Slovakia	Aa4301
	Czech Rep.	Aa4304		Aa4522	Tajekistan	Aa4283		Aa4451		Aa4399	Sweden	Aa4203				Aa4519	Czech Rep.	Aa4304
	Bulgaria	Aa4355		Aa4523		Aa4369		Aa4452		Aa4401		Aa4204						
		Aa4358		Aa4525		Aa4370		Aa4453		Aa4402	Finland	Aa4038						
						Aa4374		Aa4454		Aa4403	Romania	Aa4031						
mixed populations					Pakistan	Aa4405	Czech Rep.	Aa4304	Italy	Aa4441	Poland	Aa4190						
odd placed populations							Bulgaria	Aa4358			Czech Rep.	Aa4304						
											Slovakia	Aa4298						
											Bosnia	Aa4216						
											Slovenia	Aa4217						
											Italy	Aa4437						
												Aa4438						
												Aa4442						
												Aa4443						
												Aa4444						
												Aa4446						
												Aa4447						
												Aa4448						
												Aa4456						
											Spain	Aa4480						
												Aa4487						
											Pakistan	Aa4406						
											Bosnia	Aa4280						
											Czech Rep.	Aa4304						
											Bulgaria	Aa4355						
												Aa4358						

Figure S3. Regression of the first PC with longitude. The r = 0.93 (r^2=0.84) implies a high confidence in the linear regression. The coloured circles indicate the four groups identified in the IPGMA cluster analysis in Figure S2.

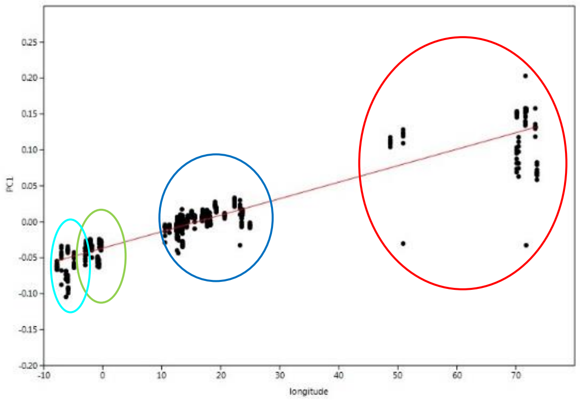

Figure S4. Pie chart showing proportion of gene ontology categories in the 51 gene models from the SNP that were identified as being under selection as defined by BayeScan and Samβada.

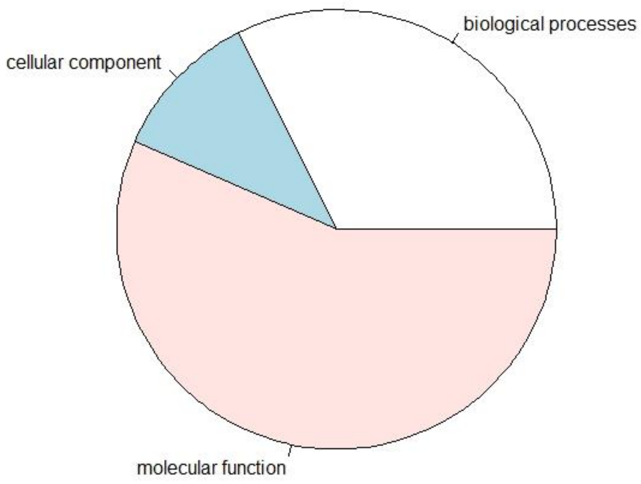

Figure S5. Comparative linkage disequilibrium decay on chromosome 1. (A): Red line=varieties, green line = ecotypes. (B): Green=UK, Red=Asia, Cyan=Iberia, Blue=Europe. (The results and decay plots were not significantly different in the other six chromosomes).

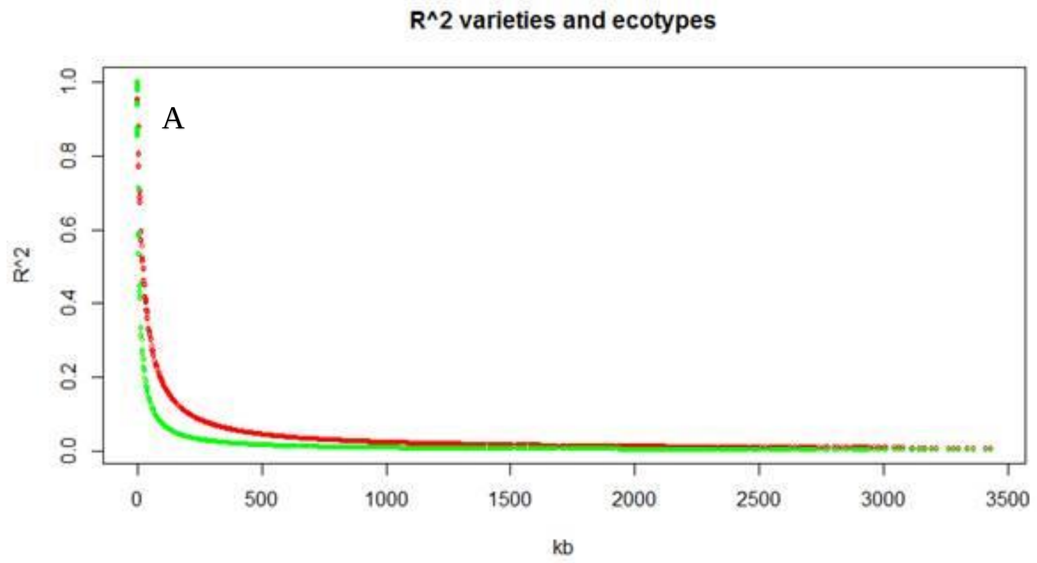

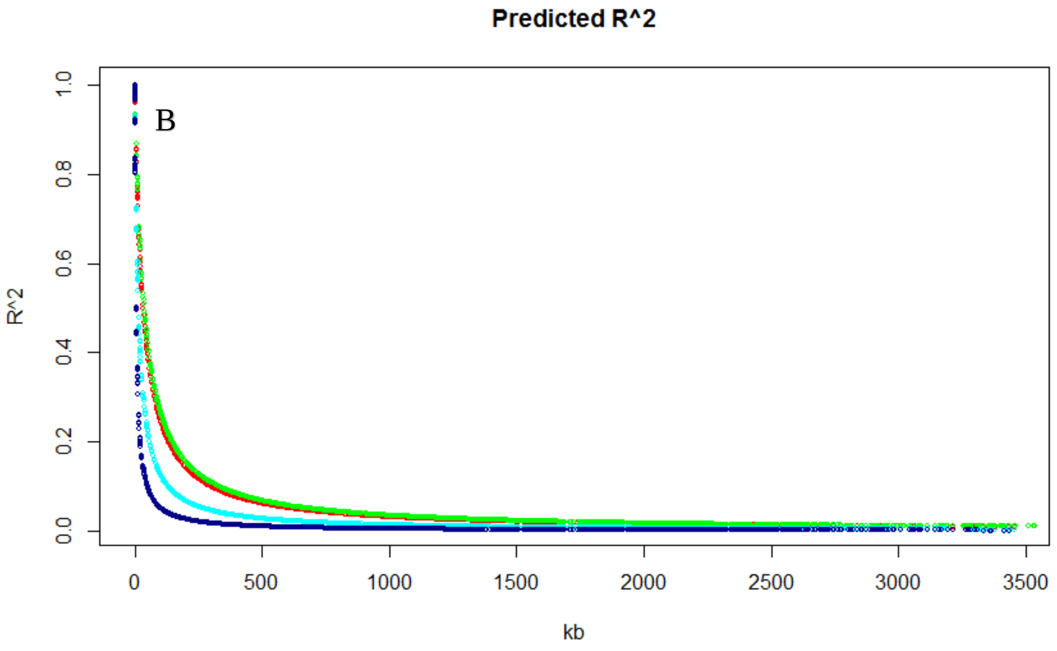

Figure S6. GWAS analysis in GAPIT using the kinship matrix and PCA covariates. **a.** QQ-plot of flowering time data, indicating two potential SNP above the significance threshold. **b.** Manhatten plot indicating a single SNP above the FDR on chromosome three.

a.

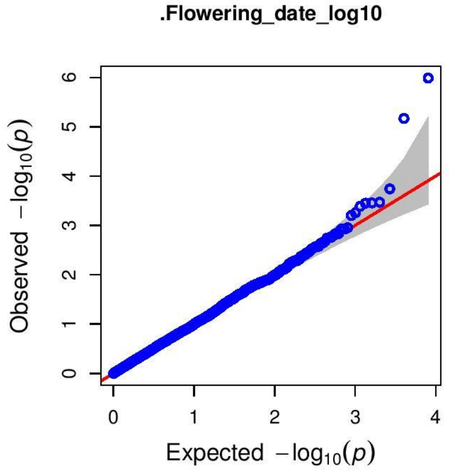

b.

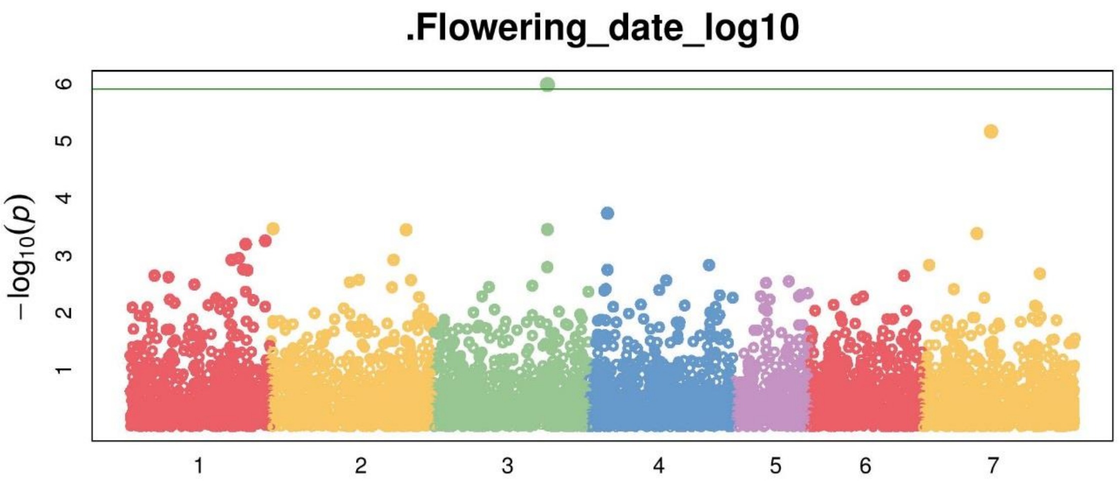

Figure S7. Average plant stem count with standard error. The data were averaged over two cuts. Measurements are in cm, and the graph is coloured to reflect the cluster analysis. The varieties are labelled AberRuby, Britta, Grasslands Broadway, Crossway, and Milvus. Average stem number ranged from 1 – 98 stems.

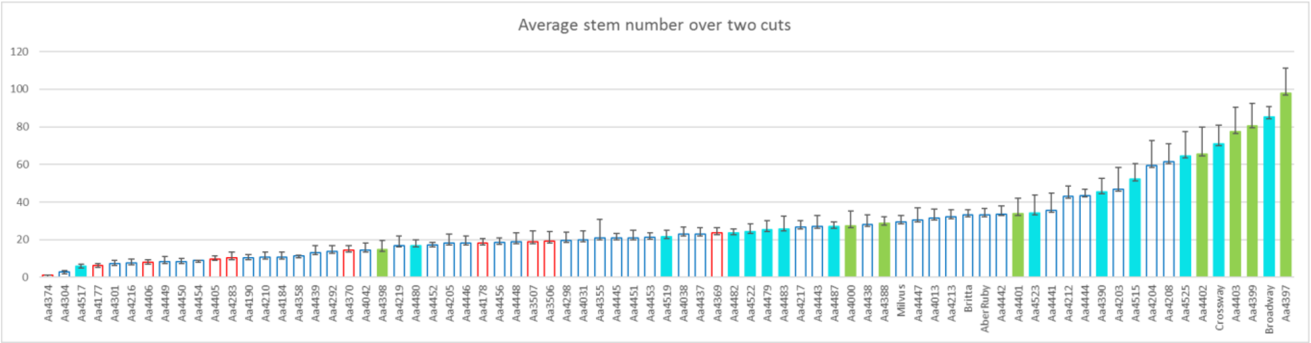

Figure S8. Region under selection in Iranian accessions Aa3506 and Aa3507. The peak describes the same genomic region on chromosome 7 in both accessions, where the F_{ST} value is above the significance threshold of 0.19 (3xSD). Genes in this region are more likely to have been under selective pressure.

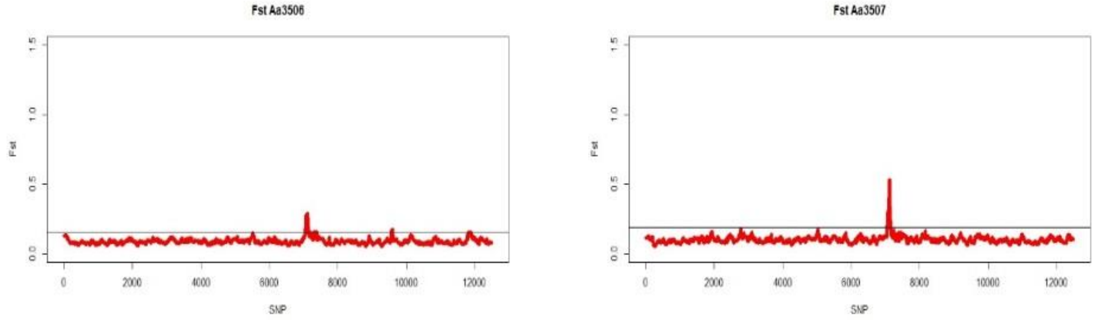

Supplement: Supplementary file 1 — Supplementary Information. [file 41598_2020_64989_MOESM1_ESM.pdf]
